# Partial tear of the medial gastrocnemius head: A case report of meniscal symptoms in a 32-year-old recreational athlete

**DOI:** 10.1007/s00113-025-01534-5

**Published:** 2025-02-06

**Authors:** Daniel P. Berthold, Fabian Traub, Fabian Gilbert, Wolfgang Böcker, Boris Holzapfel, Markus Bormann

**Affiliations:** 1 Department of Orthopaedics and Trauma Surgery, Musculoskeletal University Center Munich (MUM), University Hospital, LMU, Ziemssenstr. 5, 80336 Munich, Germany; 2 Orthocenter, Maximilianstraße 10, Munich, Germany; 3 Kreisklinikum Ebersberg, Ebersberg, Germany

**Keywords:** Knee pain, Meniscal symptoms, Recreational athlete, Magnetic resonance imaging, Conservative treatment, Knieschmerz, Typischerweise mit Meniskusverletzungen assoziierte Symptome, Freizeitsportler, Magnetresonanztomographie, Konservative Behandlung

## Abstract

This case report presents the clinical findings and management of a 32-year-old male recreational athlete who presented with ongoing knee pain for 4 months, without a history of trauma. The patient experienced intermittent pain during walking, particularly after prolonged periods of sitting, and exhibited positive findings on meniscus tests. However, he was able to participate in sports activities without pain. Magnetic resonance imaging (MRI) revealed a partial tear of the medial gastrocnemius head, confirming the diagnosis. Conservative treatment, including rest, physical therapy, and a gradual return to sports activities, led to significant symptom improvement. This case highlights the importance of considering rare injuries, such as isolated tears of the medial gastrocnemius head, in patients with persistent knee pain and meniscal symptoms, even in the absence of traumatic events. Previous reports on this specific injury are sparse, indicating its rarity and underscoring the need for further understanding and documentation.

## Abstract (de)

Der vorliegende Fallbericht beschreibt die klinischen Befunde und die Versorgung eines 32-jährigen männlichen Freizeitsportlers, der seit vier Monaten anhaltende Knieschmerzen ohne vorausgegangenes Trauma hatte. Der Patient berichtete von intermittierenden Schmerzen beim Gehen, insbesondere nach längeren Sitzphasen, und zeigte positive Befunde bei Meniskustests. Dennoch konnte er sportliche Aktivitäten schmerzfrei ausüben. Eine Magnetresonanztomographie zeigte eine Teilruptur des medialen Kopfs des M. gastrocnemius und sicherte diese Diagnose zugleich. Die konservative Behandlung, bestehend in Ruhe, Physiotherapie und einer schrittweisen Rückkehr zu sportlichen Aktivitäten, führte zu einer deutlichen Verbesserung der Symptome. Dieser Fall unterstreicht die Bedeutung der Berücksichtigung seltener Verletzungen, wie isolierter Risse des medialen Kopfs des M. gastrocnemius, bei Patienten mit anhaltenden Knieschmerzen und Symptomen, die typischerweise mit Meniskusverletzungen assoziiert sind, auch wenn sich keine traumatischen Ereignisse in der Vorgeschichte finden. Berichte über diese spezifische Verletzung sind spärlich, was auf deren Seltenheit hinweist und die Notwendigkeit betont, die Verletzung besser zu verstehen und auch besser zu dokumentieren.

## Introduction

Injuries to the gastrocnemius muscle are relatively common; however, isolated tears of the medial gastrocnemius head are particularly rare [1–4]. There is a paucity of literature and limited case reports documenting this specific injury, emphasizing its uncommon occurrence and underscoring the importance of reporting and understanding such cases. Most gastrocnemius injuries typically occur at the musculotendinous junction, predominantly manifesting as strains during activities [3, 5, 6].

The gastrocnemius muscle, consisting of two heads (medial and lateral), plays a vital role in knee flexion and ankle plantar flexion [7]. Strains and tears of the gastrocnemius muscle more commonly affect the musculotendinous junction, often resulting from sudden explosive movements, overuse, or inadequate warm-up [2]. These injuries are frequently encountered in sports activities that require rapid acceleration or involve jumping, such as basketball and sprinting [2]. However, isolated tears of the medial gastrocnemius head pose a diagnostic challenge due to their rarity and overlapping clinical presentations with other knee pathologies, such as meniscal tears.

Understanding the clinical presentation, diagnostic evaluation, and optimal management of these uncommon injuries is crucial for accurate diagnosis and appropriate treatment. This case report aims to contribute to the limited body of knowledge surrounding partial tears of the medial gastrocnemius head, particularly in the context of meniscal symptoms, by presenting the clinical findings of a 32-year-old male recreational athlete.

## Case presentation

We present the case of a 32-year-old male recreational athlete, who sought medical attention for knee pain. The patient reported experiencing intermittent knee pain during daily activities, particularly after prolonged periods of sitting, such as long flights or car drives. He engaged in recreational sports activities approximately 2–3 times per week, including running distances of 5–10 km and rowing on a rowing machine. Notably, his activities did not involve pivoting or explosive movements.

The patient denied any history of significant trauma to the knee. He had not undergone any previous injections or surgeries and had no previous knee injuries or relevant medical history. The onset of his knee symptoms was insidious (4–5 months), without any specific triggering event or identifiable cause.

The patient’s knee pain was primarily localized to the medial aspect of the joint. He did not describe occasional episodes of the knee “catching” or “locking”. Also, he reported being able to perform sports activities, including running and rowing, without experiencing any pain or discomfort.

Upon physical examination, the patient exhibited tenderness over the medial joint line. The McMurray test, as well as other medial meniscus tests, yielded positive results, further raising suspicion of a meniscal injury. However, stability testing of the knee ligaments, including the anterior cruciate ligament (ACL), posterior cruciate ligament (PCL), medial collateral ligament (MCL), and lateral collateral ligament (LCL), was negative. The patient demonstrated a full range of motion, and muscle strength was within normal limits. Notably, there was no visible joint swelling or signs of knee instability.

Given the patient’s clinical presentation, including intermittent knee pain during walking after periods of sitting, positive meniscal tests, and his involvement in sports activities without pain, a comprehensive diagnostic assessment was undertaken to further evaluate the underlying cause of his symptoms.

**Fig. 1:**
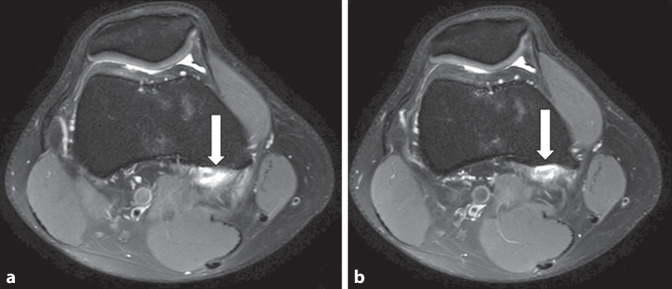
**a**, **b** Axial images indicating the partial tendon rupture of the medical gastrocnemius head

**Fig. 2:**
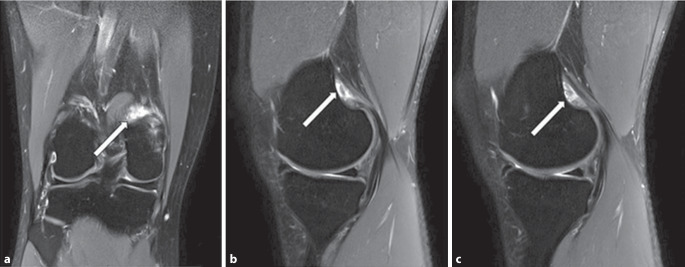
**a** Coronary and **b**, **c** sagittal images indicating the partial tendon rupture of the medial gastrocnemius head surrounding bone marrow edema

A 3 T Siemens MRI (Erlangen, Germany) (Figs. 1 and 2) was conducted.

The MRI findings confirm the presence of a partial tear at the insertion site of the medial gastrocnemius head, with associated bone marrow edema, while ruling out any significant abnormalities in the medial and lateral compartments, menisci, collateral ligaments, and biceps tendon.

## Discussion

Isolated tears of the medial gastrocnemius head are relatively rare and can pose diagnostic challenges due to their uncommon occurrence and overlapping clinical presentations with other knee pathologies, particularly medial meniscal tears [5, 6, 8]. This case report highlights the significance of considering such uncommon injuries when evaluating patients presenting with knee pain and meniscal symptoms, especially in the absence of significant trauma or previous injuries.

The medial gastrocnemius head is not commonly associated with isolated tears, with the majority of gastrocnemius injuries occurring at the musculotendinous junction [3]. In contrast, partial tears specifically involving the medial gastrocnemius head, as seen in our patient, are less frequently encountered [1, 4]. As a result, there is a limited body of knowledge and case reports available regarding the diagnosis and management of these injuries.

The clinical presentation of our patient, including intermittent knee pain during walking after periods of inactivity, positive meniscal tests, and the absence of symptoms during sports activities, initially raised suspicion of a meniscal tear [2, 9]. However, thorough diagnostic assessment, including a comprehensive physical examination and MRI, allowed for a more accurate diagnosis. The MRI findings revealed a partial tear of the medial gastrocnemius head, with associated bone marrow edema, while ruling out any significant abnormalities in the menisci, collateral ligaments, and other knee structures.

The similarity of symptoms between medial gastrocnemius head tears and medial meniscal tears underscores the importance of a comprehensive evaluation [5, 9, 10]. It is crucial for clinicians to consider less common pathologies when evaluating patients with knee pain, even when the clinical presentation initially suggests a more prevalent condition. This case report emphasizes the significance of utilizing appropriate diagnostic tools, such as MRI, to differentiate between different etiologies and ensure an accurate diagnosis [10]. In cases where a partial tear of the medial gastrocnemius head is overlooked for an extended period, there is a risk of tear progression, leading to a complete rupture. This can result in worsening pain, significant functional weakness, and prolonged recovery times, potentially impairing mobility and daily activities.

Conservative management was chosen for our patient, considering his young age, absence of significant trauma, and stable knee ligaments [5, 8, 10]. A tailored physical therapy program focusing on strengthening the surrounding muscles, improving flexibility, and proprioception training yielded favorable outcomes. At the 6‑month follow-up, the patient reported significant improvement in symptoms, with no pain during walking or participation in sports activities. Physical examination findings were also reassuring, demonstrating the effectiveness of conservative management in this particular case.

## Conclusion

Isolated partial tears of the medial gastrocnemius head are uncommon injuries that can mimic the symptoms of medial meniscal tears [1–4, 9]. A high index of suspicion is required to consider this diagnosis, especially in cases with atypical clinical presentations or inconclusive physical examination findings. Diagnostic tools such as MRI play a crucial role in accurately identifying the underlying pathology [5, 8–10]. Conservative management, including physical therapy, can lead to favorable outcomes, enabling patients to return to their desired level of athletic participation [2, 5, 9, 10]. Further research and case reports are necessary to enhance our understanding of this rare injury and its optimal management strategies.

## Declarations

The authors declare that no Institutional Review Board (IRB) approval was required, as this work does not constitute a study. All studies mentioned were in accordance with the ethical standards indicated in each case. For images or other information within the manuscript which identify patients, consent was obtained from them and/or their legal guardians.
